# Long-Term SARS-CoV-2 Infection Associated with Viral Dissemination in Different Body Fluids Including Bile in Two Patients with Acute Cholecystitis

**DOI:** 10.3390/life10110302

**Published:** 2020-11-23

**Authors:** Rossana Scutari, Lorenzo Piermatteo, Matteo Ciancio Manuelli, Marco Iannetta, Romina Salpini, Ada Bertoli, Claudia Alteri, Patrizia Saccomandi, Maria Concetta Bellocchi, Vincenzo Malagnino, Elisabetta Teti, Daniele Sforza, Leandro Siragusa, Michele Grande, Loredana Sarmati, Valentina Svicher, Massimo Andreoni, Francesca Ceccherini-Silberstein

**Affiliations:** 1 Department of Experimental Medicine, University of Rome “Tor Vergata”, 00133 Rome, Italy; scutari.rossana@gmail.com (R.S.); lorenzo.piermatteo@uniroma2.it (L.P.); rsalpini@yahoo.it (R.S.); bertoli@uniroma2.it (A.B.); patriziasaccomandi22@hotmail.com (P.S.); mariac.bellocchi@gmail.com (M.C.B.); ceccherini@med.uniroma2.it (F.C.-S.); 2 Surgical Emergency Unit, Emergency Department, Polyclinic Tor Vergata Foundation, 00133 Rome, Italy; matt.manuelli@gmail.com (M.C.M.); dsforza@gmail.com (D.S.); 3 Department of System Medicine, University of Rome “Tor Vergata”, 00133 Rome, Italy; marco.iannetta@uniroma2.it (M.I.); malagninovincenzo@gmail.com (V.M.); elisabetta.teti@gmail.com (E.T.); sarmati@med.uniroma2.it (L.S.); andreoni@uniroma2.it (M.A.); 4 Laboratory of Clinical Microbiology and Virology, Polyclinic Tor Vergata Foundation, 00133 Rome, Italy; 5 Department of Oncology and Hemato-oncology, University of Milan, 20122 Milan, Italy; claudia.alteri@unimi.it; 6 Department of Surgical Sciences, University of Rome “Tor Vergata”, 00133 Rome, Italy; leandros93@hotmail.it (L.S.); grande@uniroma2.it (M.G.)

**Keywords:** SARS-CoV-2, RNA quantification, droplet digital PCR, RT-PCR, body compartment

## Abstract

Our study aimed to investigate the kinetics of SARS-CoV-2 RNA in bile and in different body fluids of two SARS-CoV-2 positive patients with acute cholecystitis by innovative droplet digital PCR (ddPCR) assays. For each patient, nasopharyngeal- and rectal swabs, bile, urine, and plasma samples were collected at different time points for SARS-CoV-2 RNA quantification by two ddPCR assays. For both patients, ddPCR revealed persistent and prolonged detection of viral RNA in the nasopharyngeal swab despite triple-negative or single-positive results by qRT-PCR. In Patient 1, SARS-CoV-2 RNA dropped more rapidly in bile and rectal-swab and declined slowly in nasopharyngeal swab and plasma, becoming undetectable in all compartments 97 days after symptoms started. Conversely, in patient 2, SARS-CoV-2 RNA was detected, even if at low copies, in all body samples (with the exception of urine) up to 75 days after the onset of symptoms. This study highlights that SARS-CoV-2 RNA can persist for a prolonged time in respiratory samples and in several biological samples despite negativity to qRT-PCR, supporting SARS-CoV-2’s ability to provoke persistent and disseminated infection and therefore to contribute to extra-pulmonary clinical manifestations.

## 1. Introduction

At the end of 2019, a new coronavirus, known as SARS-CoV-2 (Severe Acute Respiratory Syndrome Coronavirus 2), emerged in China [1,2,3], spread worldwide and, on 11 March 2020, the WHO publicly declared the SARS-CoV-2 outbreak as a pandemic [2,4].

Clinically, SARS-CoV-2 infection ranges from asymptomatic carrier status to a long-lasting and severe lower respiratory tract infection [5]. Despite the fact that the respiratory tract is the principal site of infection and viral replication, there is growing evidence of the persistence of viral particles in extra-pulmonary districts [6,7,8,9,10]. Notably, SARS-CoV-2 has been detected in feces, peritoneal fluid and cells lining the gastrointestinal tract [9,11], suggesting the role of the digestive system as a route of infection. This is further supported by recent studies showing the expression of the SARS-CoV-2 Angiotensin-converting enzyme 2 (ACE2) receptor on the cell surface of absorptive enterocytes from the ileum and colon [12].

In clinical practice, the gold standard for the detection and quantification of SARS-CoV-2 RNA is the real-time reverse transcriptase polymerase chain reaction (qRT-PCR). Nevertheless, this method has been questioned due to low sensitivity and specificity [13].

Recently, attention has been focused on the use of the droplet digital PCR (ddPCR) system for the quantification of SARS-CoV-2 RNA. This assay provides a reliable absolute quantification of viral RNA and is endowed with a higher sensitivity compared to qRT-PCR, showing a particular usefulness for quantifying low viral load samples [14,15,16].

In this light, we investigated the detectability and kinetics of SARS-CoV-2 RNA in different body fluids, including the bile, of two SARS-CoV-2 positive patients with acute cholecystitis, treated conservatively with a cholecystostomy.

## 2. Materials and Methods 

### 2.1. Patients and Clinical Samples Collection

This study was conducted at the “Tor Vergata” University Hospital in collaboration with the Virology research laboratory of the University of Rome “Tor Vergata”. Two patients with confirmed SARS-CoV-2 infection hospitalized and with a peculiar clinical history were investigated.

Demographic data (age, gender, underlying diseases) and clinical history (initial symptoms or signs associated with SARS-CoV-2 infection, treatment regimen, duration from admission to resolution of symptoms and signs) were collected for both patients.

Furthermore, for each patient, nasopharyngeal and rectal swabs, urine, bile and plasma samples were collected at different time points during their hospitalization. In particular, samples were collected from 12 May to 2 July (51 days monitoring) for patient 1, and from 28 May to 10 June (13 days monitoring) for patient 2. The interval between the diagnosis of SARS-CoV-2 infection and the start of sample collection was 46 and 63 days for patient 1 and 2, respectively. Both patients were brought to our attention by another Roman hospital.

### 2.2. Ethical Committee 

The study protocol was approved by local Research Ethics Committee (prot. number 46.20) and conducted in accordance with the principles of the 1964 Declaration of Helsinki. Both patients signed informed consents. Furthermore, the research was conducted on viral samples and data previously anonymized, according to the requirements of the Italian Data Protection Code (leg. decree 196/2003). 

### 2.3. SARS-CoV-2 RNA Quantification 

#### 2.3.1. Real Time-PCR

qRT-PCR was used for the quantification of SARS-CoV-2 RNA in nasopharyngeal swabs at different time points, during hospitalization at the University Hospital Tor Vergata, for diagnostic purposes. The GeneFinderTM COVID-19 Plus RealAmp Kit, ELITech AllplexTM 2019-nCoV Assay (Seegene) used for Real Time-PCR was based on the identification of three genetic viral targets: E, RdRp and N genes. The cycle threshold (Ct) obtained from qRT-PCR was used as measure of viral load and is inversely proportional to viral load values: lower Ct corresponds to higher viral loads, and, conversely, higher Ct levels indicate lower viral loads.

#### 2.3.2. ddPCR

ddPCR was used for the quantification of SARS-CoV-2 RNA in nasopharyngeal swabs and in the other biological samples (rectal swabs, urine, bile and plasma samples) collected.

Total RNA was extracted from 280 µL of all clinical samples by using the QIAamp viral RNA mini kit (Qiagen, Hilden, Germany) according to manufacturer’s instruction. SARS-CoV-2 RNA was quantified by QX200™ Droplet Digital™ PCR System (ddPCR, Biorad, Hercules, CA, USA) using a home-made assay, targeting the RdRp gene of SARS-CoV-2 as previously described. The assay also targets the housekeeping gene RNAse P as internal control of amplification. The following formula was applied to express the result in copies/1 mL of biological sample:

copies of eluted RNA:0.28 mL = X:1 mL

where 0.28 mL is the extraction starting volume.

Moreover, to confirm our results, the commercial triplex ddPCR assay (2019-nCoV CDC ddPCR Triplex Probe Assay, Assay ID: dEXD28563542) kindly provided by Bio-Rad was used according to manufacturer’s instructions. Specifically, this assay is based on the recognition of three targets, two for the viral nucleocapsid gene and one for the human RPP30 gene as reaction control.

## 3. Results

### 3.1. Patient

#### 3.1.1. Clinical and Surgical History

An 80-year-old Caucasian man after acute onset of fever, myalgia and cough in a rehabilitation clinic, where he was recovering from a recent operation for a hip replacement, was diagnosed with SARS-CoV-2 infection after a positive nasal swab on 27 March 2020. After performing a chest computed tomography (CT) scan, which showed the presence of bilateral pneumonia and pleural effusion, he was soon hospitalized in a COVID-19 dedicated center. The patient was a non-smoker and had a history of hypertension and benign prostatic hyperplasia.

On 5 April 2020 the patient underwent orotracheal intubation and mechanical ventilation because of respiratory failure due to worsening of pneumonia and pleural effusion. SARS-CoV-2 infection was treated for 10 days with 4-hydroxychloroquine, remdesivir and with one infusion of sarilumab and the patient was extubated on 13 April 2020.

On 8 May 2020 the patient underwent an abdomen CT-scan for acute abdominal pain showing a 12.5 × 6 cm hydropic gallbladder with wall thickening, a 2.7 cm gallstone in the infundibulum and pericholecystic reactive fluid. Two days later a cholecystostomy was placed and Enterococcus faecalis was isolated by biliary culture; the patient was then transferred to the surgery department. The presence of the cholecystostomy allowed the periodic sampling of bile for routine microbiological exams and SARS-CoV-2 molecular tests. Patient’s clinical course was complicated by the occurrence of a 6.5 × 3.5 cm spontaneous left iliopsoas muscle hematoma (treated with the radiological guided placement of a drainage) and right external iliac vein thrombosis (managed with low-molecular weight heparin and the placement of an inferior vena cava (IVC) filter).

Patient was discharged in good clinical condition on 14 June 2020 with cholecystostomy and IVC filter still in place and was periodically monitored in the surgical outpatient clinic.

Hematological parameters and inflammation markers during hospitalization are summarized in Supplementary Table S1.

#### 3.1.2. Virological Characterization

On 12 May (the first time point of sample collection, two days after cholecystostomy, 46 days after symptoms started and 30 days after the ending of treatment with antivirals) both ddPCR assays allowed the quantification of SARS-CoV-2 RNA in all samples with the exception of urine (Table 1). As expected, the highest RNA load was observed in nasopharyngeal swabs (594 and 266 copies/mL, each assay, respectively), while the lowest levels were observed in the rectal swabs (22 and 52 copies/mL, each assay, respectively). Notably, SARS-CoV-2 RNA was detected in plasma and in bile with a comparable viral RNA load, suggesting a potential disseminated infection (Table 1). The day after, SARS-CoV-2 RNA load tended to decay in the nasopharyngeal swab, in line with the single-target positive result, with low Ct by qRT-PCR (Ct = 38.5 for N gene). At the following three time points during hospitalization, (on 19 May, 26 May and 10 June, 53, 60 and 75 days after symptoms started, respectively), RNA load in nasopharyngeal swabs continued to be intermittently detected by ddPCR for more targets (although at low levels) despite completely negative results obtained with qRT-PCR (Table 1).

Similarly, RNA load in plasma samples and rectal swabs underwent a progressive decrease, although remaining persistently detected up to four weeks after the virological follow-up (10 June, Table 1). In bile, SARS-CoV-2 RNA was detected for at least one week (up to 19 May) and then was undetectable (Table 1). Conversely, SARS-CoV-2 RNA was never detected in urine at all time points analysed.

On 2 July, during a surgical control in the outpatient clinic, SARS-CoV-2 RNA was completely negative in the above-mentioned biological samples analysed, supporting resolved SARS-CoV-2 infection after 97 days of follow up (Table 1).

### 3.2. Patient 2

#### 3.2.1. Clinical and Surgical History

A 74-year-old Caucasian man presented to the emergency department on 26 March 2020 with acute respiratory distress and a positive SARS-CoV-2 RNA nasopharyngeal swab. Patient was a former smoker and had an history of hypothyroidism, paroxysmal atrial fibrillation and hypertension. After experiencing respiratory failure, the patient required tracheostomy and admission in the intensive care unit for invasive ventilation. The patient was treated for 10 days with darunavir, ritonavir and 4-hydroxychloroquine with gradual improvement of lung function and the patient was weaned from mechanical ventilation on 9 April 2020.

On 7 May 2020 the patient presented acute abdomen with right hypochondrium tenderness and positive Murphy sign. An abdomen CT-scan revealed an acute cholecystitis with hydropic gallbladder presenting diffuse wall thickening and containing biliary sludge. Patient was referred to surgeon for cholecystostomy.

On 13 May 2020, given the clinical patient’s characteristic, the patient underwent a cholecystostomy.

Post-procedure clinical course was regular and followed by full resolution of cholecystitis and pneumonia. On 16 June 2020 the cholecystostomy was removed and patient was discharged in good clinical condition.

Hematological parameters and inflammation markers during hospitalization are summarized in Supplementary Table S2.

#### 3.2.2. Virological Characterization

At the first time point of sample collection (on 28 May, 63 days after symptoms started and 49 days after the ending of treatment with antivirals), SARS-CoV-2 RNA was quantified in all biological samples (including bile and urine) (Table 2). As expected, the highest RNA load was observed in nasopharyngeal swabs, followed by the rectal swabs.

By both ddPCR assays, SARS-CoV-2 RNA in nasopharyngeal swabs was quite stable in the first seven days of evaluation (574–818 copies/mL one assay, 214–244 copies/mL second assay) and underwent a drastic drop after 13 days in line with the results by qRT-PCR (triple-target positive result after seven days and double-target positive result after 13 days) (Table 2). Similarly, a progressive decline of SARS-CoV-2 RNA was observed in rectal swabs by ddPCR assays during the time-points analysed. Conversely, both ddPCR assays revealed a stable trend over time of SARS-CoV-2 RNA in plasma and bile although at low level. In urine, SARS-CoV-2 RNA was present at low level only at the first time point of sample collection (Table 2).

## 4. Discussion

This study reports two clinical cases characterized by long-term SARS-CoV-2 RNA detection in multiple biological fluids, including bile, both affected by acute cholecystitis and treated conservatively with a cholecystostomy.

The use of “ad hoc” ddPCR assays have allowed a better unraveling the kinetics of viral persistence and dissemination over time. In particular, both patients showed positivity to SARS-CoV-2 RNA up to 75 days after the symptoms’ onset despite negative or double-positive results by RT-PCR, respectively. Such viral RNA positivity was observed not only in the upper respiratory tract but also in different anatomical compartments.

Several studies have highlighted the persistence of SARS-CoV-2 in the respiratory region for a long time despite the resolution of clinical symptoms [17,18,19]. In particular, Cento et al. confirmed prolonged RT-PCR positivity in a significant proportion of clinically recovered patients (46.9% at clinical recovery, 13.7% at day 14 after hospital discharge, and 14.7% between day 41 and day 60 after hospital discharge) [18].

These results highlight the need for a long-term virological follow-up of SARS-CoV-2 infected patients despite resolution of major clinical symptoms to confirm the clearance of SARS-CoV-2 infection. Furthermore, long-term SARS-CoV-2 infection also raises the importance of a better unraveling of the significance of this positivity in term of virus transmissibility.

Beyond nasopharyngeal swabs, SARS-CoV-2 RNA was detected in different biological fluids, including plasma, urine and rectal swabs, although a potential contamination from the upper respiratory tract cannot be excluded. Notably, in our real life experience, we have currently analysed a small group of rectal swabs and we found that 20/28 were SARS-CoV-2 RNA positive, generally at low levels, but in two cases also with values >1000 copies/mL (unpublished data). These results are in line with other studies and support viral potential to spread to extra-pulmonary districts and to give origin to disseminated infection that may explain the large spectrum of clinical manifestations associated with SARS-CoV-2 infection [8,14,20,21].

Notably, this study provides evidence of persistent SARS-CoV-2 detection in bile, in line with a recent report by Deheng et al. [10]. The viral receptors ACE-2 are expressed in the gastrointestinal tract, so the presence of SARS-CoV-2 RNA in bile fluid can be attributed to the bile recirculation process. Furthermore, and more intriguingly, SARS-CoV-2 RNA in bile can be also explained by the flow of bile through biliary ducts whose epithelium is composed by ACE-2-expressing cholangiocytes. This raises the possibility for SARS-CoV-2 entering and replicating in these cells, injuring cholangiocytes [22], thus supporting a direct role of SARS-CoV-2 in pathobiological mechanisms underlying cholecystitis and cholestasis. In line with this concept, in Patient 1 the first bile sample analyzed was collected just four days after the onset of cholecystitis.

There is evidence of viral replication in gallbladder epithelial cells, which are very similar to bile duct cells and express ACE-2, being a permissive target of SARS-CoV-2 infection [23].

The rapid decline of SARS-CoV-2 viral load in bile juice after gallbladder drainage in patient 1, was probably due to the progressive replacement of the bile accumulated in the days before the drainage with newly produced fluid, once the obstruction was removed. Moreover, the persistence of SARS-CoV-2 viral detection up to 10 days after the drainage was probably due a residual active replication of the virus in the bile duct and gallbladder epithelial cells. In the following assessments, SARS-CoV-2 RNA was no longer detected in bile juice, demonstrating the cessation of viral replication in the gallbladder and biliary ducts and corresponding to the clinical improvement of the patient. Concerning patient 2, bile juice sampling was performed 15 days after gallbladder drainage, when the fluid was consistently replaced by newly produced fluid. Consequently, in this case we could not observe a dramatic decrease in SARS-CoV-2 RNA viral load in the bile fluid.

DdPCR has allowed the quantification of SARS-CoV-2 RNA in a large variety of biological specimens, also in the setting of triple-target negative or single-target positive results by qRT-PCR. Both ddPCR assays used in our study have allowed the persistent detection of SARS-CoV-2 RNA over time, even though at low copies. Although persistence of viral RNA cannot be associated with disease severity, it may reflect an impaired capability of the immune system to promote virus RNA clearance. 

Our study has some limitations: two patients analyzed for different time points and with different characteristics; the real infectivity of the virus found in the different compartments was not assessed, and the presence of viral RNA does not distinguish between infectious and non-infectious virus.

Overall, these findings support the role of ddPCR for clinical detection and quantification of SARS-CoV-2 RNA, in reducing false negative results, thus representing a valuable complement to the current standard RT-PCR.

In conclusion, our study highlights that SARS-CoV-2 RNA can persist for a prolonged time in respiratory samples and in several biological samples despite negativity to qRT-PCR, corroborating SARS-CoV-2’s potential to give origin to persistent and disseminated infection that could pose the basis for extra-pulmonary clinical manifestations and delayed clearance of the infection.

## Figures and Tables

**Table 1:** Results of SARS-CoV-2 RNA quantification over time for patient 1.

Date of Sample Withdrawal	12 May			13 May			19 May			26 May			10 June			2 July		
Days after respiratory symptoms start		46			47			53			60			75			97	
Gene targeted by assays for RNA quantification	N	RdRp	E	N	RdRp	E	N	RdRp	E	N	RdRp	E	N	RdRp	E	N	RdRp	E
Results by ddPCR ^a^																		
Nasopharyngeal swab, copies/mL	594	266	n.a.	247	94	n.a.	46	TND	n.a.	8	10	n.a.	10	17	n.a.	TND	TND	n.a.
Plasma, copies/mL ^b^	84	176	n.a.	-	-	-	TND	30	n.a.	6	11	n.a.	6	11	n.a.	TND	TND	n.a.
Bile, copies/mL ^b^	100 *	86 *	n.a.	6	29	n.a.	18	27	n.a.	-	-	-	TND	TND	n.a.	TND	TND	n.a.
Rectal swab, copies/mL ^b^	22	52	n.a.	-	-	-	6	10	n.a.	TND	12	n.a.	TND	TND	n.a.	TND	TND	n.a.
Urine, copies/mL ^b^	TND	TND	n.a.	-	-	-	TND	TND	n.a.	TND	TND	n.a.	TND	TND	n.a.	-	-	-
Ct obtained by Real-time PCR ^a^																		
Nasopharyngeal swab ^b^	-	-	-	38.5	Neg	Neg	Neg	Neg	Neg	Neg	Neg	Neg	Neg	Neg	Neg	-	-	-

^a^ Two droplet digital PCR assays were used: the former targeting the gene encoding nucleocapsid was developed by Bio-Rad; the latter targeting RdRp was home made. Real-Time PCR targeted three genes encoding nucleocapsid, RdRp and the Envelope protein. The Ct reported in the table have been obtained at most 24–48 h respect to the timepoints reported in the table. ^b^ (–) indicates lack of biological sample at that specific time point. * SARS-CoV-2 RNA at two days after cholecystostomy. Abbreviations: n.a.: not applicable; TND: Target not detected; N: nucleocapsid; RdRp: RNA-dependent RNA polymerase, E: envelope protein; ddPCR: droplet digital polymerase chain reaction; Ct: cycle threshold.

**Table 2:** Results of SARS-CoV-2 RNA quantification over time for patient 2.

Date of Sample Withdrawal	28 May			4 June			10 June		
Days after respiratory symptoms start		63			70			75	
Gene targeted by assays for viral RNA quantification	N	RdRp	E	N	RdRp	E	N	RdRp	E
Results by ddPCR ^a^									
Nasopharyngeal swab, copies/mL	574	214	n.a.	818	244	n.a.	16	17	n.a.
Plasma, copies/mL	11	27	n.a.	12	11	n.a.	6	11	n.a.
Bile, copies/mL	12 *	20 *	n.a.	12	11	n.a.	19	29	n.a.
Rectal swab, copies/mL	220	43	n.a.	14	14	n.a.	5	23	n.a.
Urine, copies/mL	12	31	n.a.	TND	TND	n.a.	TND	TND	n.a.
Ct obtained by Real-time PCR ^a^									
Nasopharyngeal swab	25.8	28.5	26.9	31.2	31.1	29.6	38.3	29.7	Neg

^a^ Two droplet digital PCR assays were used: the former targeting the gene encoding nucleocapsid was developed by Bio-Rad; the latter targeting RdRp was home made. Real-Time PCR targeted three genes encoding nucleocapsid, RdRp and the Envelope protein. The Ct reported in the table have been obtained at most 24–48 h respect to the timepoints reported in the table. * SARS-CoV-2 RNA at 15 days after cholecystostomy. Abbreviations: n.a.: not applicable; TND: Target not detected; N: nucleocapsid; RdRp: RNA-dependent RNA polymerase, E: envelope protein; ddPCR: droplet digital polymerase chain reaction; Ct: cycle threshold.

## Supplementary Materials

The following are available online at https://www.mdpi.com/2075-1729/10/11/302/s1, Table S1. Hematological parameters and inflammatory markers for patient 1; Table S2. Hematological parameters and inflammatory markers for patient 2.
