# Supplementary material for: Long-Term SARS-CoV-2 Infection Associated with Viral Dissemination in Different Body Fluids Including Bile in Two Patients with Acute Cholecystitis
Source: Life (Basel). 2020 Nov 23;10(11):302. doi: 10.3390/life10110302 (PMC7700357; doi:10.3390/life10110302)
Supplement: Supplementary file 1 [file life-10-00302-s001.pdf]

life

MDPI

Supplementary Materials

Long-Term SARS-CoV-2 Infection Associated with Viral Dissemination in Different Body Fluids Including Bile in Two Patients with Acute Cholecystitis

Table S1. Hematological parameters and inflammatory markers for patient 1.

**Date of sample withdrawal**	**May 12th**	**May 13th**	**May 19th**	**May 26th**	**June 10th**	**July 2nd**
White blood cells (cells/μL)	6.930	6.340	6.280	6.760	6.180	6.370
Neutrophils (cells/μL)	5.130	4.320	4.250	3.410	4.380	4.560
Lymphocyte (cells/μL)	1.290	1.370	1.460	2.480	1.480	1.350
N/L ratio	3.98	3.15	2.91	1.38	2.96	3.38
CRP (mg/L) (normal range: 0–5)	55.8	16.3	11.9	35.7	2.8	26.7
IL-6 (pg/L) (normal range: <50)	53.0	n.a.	4.61	21.8	5.6	13.4
TNF-alpha (pg/L) (normal range: 4.6–12.4)	9.80	n.a.	9.51	28.28	8.13	16.59
D-dimers (ng/L) (normal range: 0–500)	3617	n.a.	1318	979	3657	n.a.

N/L ratio: Neutrophils to Lymphocyte ratio; CRP: C-Reactive protein; IL-6: interleukin 6; TNF-alpha: Tumor necrosis factor-alpha; n.a.: not available.

Table S2. Hematological parameters and inflammatory markers for patient 2.

**Date of sample withdrawal**	**May 28th**	**June 4th**	**June 10th**
White blood cells (cells/μL)	11.380	3.080	10.160
Neutrophils (cells/μL)	4.250	2.160	2.210
Lymphocyte (cells/μL)	5.970	5.730	6.710
N/L ratio	0.71	0.38	0.33
CRP (mg/L) (normal range: 0–5)	7.3	5.9	3.2
IL-6 (pg/L) (normal range: <50)	25.3	n.a.	11.6
TNF-alpha (pg/L) (normal range: 4.6–12.4)	58.86	n.a.	35.3
D-dimers (ng/L) (normal range: 0–500)	655	809	917

N/L ratio: Neutrophils to Lymphocyte ratio; CRP: C-Reactive protein; IL-6: interleukin 6; TNF-alpha: Tumor necrosis factor-alpha; n.a.: not available.

Publisher's Note: MDPI stays neutral with regard to jurisdictional claims in published maps and institutional affiliations.

© 2020 by the authors. Submitted for possible open access publication under the terms and conditions of the Creative Commons Attribution (CC BY) license (http://creativecommons.org/licenses/by/4.0/).
